# Association between fluid intake and mortality in critically ill patients with negative fluid balance: a retrospective cohort study

**DOI:** 10.1186/s13054-017-1692-3

**Published:** 2017-05-12

**Authors:** Yanfei Shen, Xinmei Huang, Weimin Zhang

**Affiliations:** 1 Department of Intensive Care Unit, Dongyang People’s Hospital, No. 60, Wuning West Road, Dongyang, Zhejiang 322100 People’s Republic of China; 2 Department of otolaryngological, Jinhua TCM hospital, No. 439, Shuangxi West Road, Jinhua, Zhejiang 322100 People’s Republic of China

**Keywords:** Critically ill, Negative fluid balance, Fluid intake, Diuretics, Mortality

## Abstract

**Background:**

Compared to positive fluid balance (FB), negative FB is associated with improved clinical outcomes in critically ill patients. However, as to whether achieving more negative FB can further improve outcomes has not been investigated. This study aimed to investigate whether more negative FB and restricted fluid intake were associated with improved outcomes in critically ill patients.

**Method:**

Data were extracted from the Multi-parameter Intelligent Monitoring in Intensive Care III Database. Patients achieving negative FB at 48 hours after intensive care unit (ICU) admission were screened. The primary outcome was hospital mortality. Logistic models were built to explore the association between FB, fluid intake and mortality, using FB and fluid intake (both four levels) as design variables and using the linear spline function method.

**Results:**

There were 2068 patients meeting the inclusion criteria. Compared to slight negative FB (level 1), there was a decreased tendency towards mortality with FB level 2 (OR 0.88, 95% CI 0.69–1.11) and level 3 (OR 0.79, 95% CI 0. 65–1.11); however, only extreme negative FB (level 4) was significant (OR 0.56, 95% CI 0. 33–0.95). Fluid intake and urine output were evenly distributed over the first 48 hours after ICU admission. Fluid intake was inversely associated with hospital mortality, with the OR decreased stepwise from level 2 (OR 0.73, 95% CI 0.56–0.96) to level 4 (OR 0.47, 95% CI 0.30–0.74), referred to level 1. Urine output also showed a similar pattern. Diuretic use was associated with higher mortality in both models.

**Conclusion:**

In critically ill patients with negative FB, both increased fluid intake and urine output were associated with decreased hospital mortality. However, compared to slight FB, achieving more negative FB was not associated with reduced mortality.

**Electronic supplementary material:**

The online version of this article (doi:10.1186/s13054-017-1692-3) contains supplementary material, which is available to authorized users.

## Background

Appropriate fluid management in critically ill patients is one of the most challenging and difficult aspects of care for the patient care team. Despite the fact that sufficient fluid resuscitation is important for stabilizing hemodynamic status and improving tissue oxygenation, positive correlation between fluid overload and adverse outcomes has been proven in critically ill patients. Several studies have indicated that positive cumulative fluid balance (FB) is a strong prognostic factor for mortality in sepsis [1, 2]. An analysis of the vasopressin and septic shock trial [3] also showed that a more positive FB at 12 hours and cumulatively over 4 days is associated with increased risk of mortality. Similar results were also reported in other specific patient populations, such as those with acute renal failure [4], acute lung injury [5], aneurysmal subarachnoid hemorrhage (ASH) [6] and surgical patients [7].

Based on the current evidence, conservative fluid administration and diuretics were widely adopted in these populations to achieve earlier and more negative FB once the hemodynamic status was stable. However, most of the current studies focused on the comparison between positive and negative FB; whether achieving more negative FB could further improve outcomes in critically ill patients has not been investigated.

Aside of this, research has also indicated that once adequate fluid resuscitation is achieved, further fluid administration may increase intravascular pressure along with vascular permeability, causing fluid leakage resulting in tissue edema, decreased oxygenation index, higher incidence of acute kidney injury (AKI) [8] and increased mortality [3, 9]. However, in patients with negative FB, fluid administration is less likely to further aggravate fluid overload. Thus, whether restricted fluid management still applies to this population remains uncertain. The purpose of this study was to investigate whether more negative FB and restricted fluid intake are still associated with improved outcomes in critically ill patients with negative FB.

## Methods

### Data source

The Multi-parameter Intelligent Monitoring in Intensive Care (MIMIC) III (version 1.4) database is maintained by the Laboratory for Computational Physiology at the Massachusetts Institute of Technology (Cambridge, MA, USA). It contains more than 40,000 ICU patients attending Beth Israel Deaconess Medical Center (Boston, MA, USA) from 2001 to 2012 [10, 11]. The database is accessible to researchers who have passed “protecting human subjects training”. The institutional review boards of the Massachusetts Institute of Technology and Beth Israel Deaconess Medical Center approved the establishment of the database and all the information used was anonymous. Therefore, the ethical approval statement and informed consent was waived for this manuscript. Data were extracted by author YS (certification number: 1564657).

### Study population and stratification

This study aimed to explore appropriate fluid management in critically ill patients with negative FB. Patients who were older than 18 years and stayed in ICU more than 48 hours were screened for inclusion. For patients with more than one ICU stay, only the first ICU stay was eligible for inclusion. Only patients who achieved negative FB at 48 hours after ICU admission were further analyzed. Patients who underwent cardiac surgery or any form of renal replacement therapy were excluded due to the great impact on FB of these procedures.

Fluid intake, fluid output and urine output (UO) were recorded at 48 hours after ICU admission. FB was calculated as fluid intake minus fluid output. For interpretation, fluid intake, UO and FB were used as design variables in logistic regression. We also stratified fluid intake, UO and FB by increasing the volume instead of using a quartile approach, as the aim of the current study was to explore the impact of the volume of fluid intake and FB on mortality. Thus, fluid intake and UO were categorized into four levels, using 30 ml/kg/48 hours as one interval: level 1 (≤29 ml/kg/48 hours), level 2 (30 ~ 59 ml/kg/48 hours), level 3 (60 ~ 89 ml/kg/48 hours) and level 4 (>90 ml/kg/48 hours); FB was classified using 20 ml/kg/48 hours as one interval: level 1 (−19 ~ 0 ml/kg/48 hours), level 2 (–39 ~ –20 ml/kg/48 hours), level 3 (–59 ~ −40 ml/kg/48 hours) and level 4 (≤ −60 ml/kg/48 hours). Sensitivity analysis was performed after including patients without a disease severity score.

### Definitions and outcomes

The primary endpoint was hospital mortality. Secondary endpoints included hospital length of stay (LOS), ICU LOS, AKI incidence, and maximum sequential organ failure assessment (SOFA) and simplified acute physiology score (SAPS) II during the ICU stay. A rise of more than 1.5 times above baseline serum creatinine was considered as AKI according to the creatinine-based Kidney Disease Improving Global Outcome criteria [12]. Loop diuretics and blood products use were defined as any diuretics or blood products used within 48 hours of ICU admission for any reason.

### Statistical analysis

Continuous variables were presented as mean and standard error (SE) or median and interquartile range (IQR) as appropriate. Student’s *t* test or analysis of variance (ANOVA), or the Wilcoxon rank-sum test or Kruskal-Wallis test was used as appropriate. Categorical variables were presented as a percentage and compared using the chi-square test [13]. Lowess smoother technique was used to explore the crude relationship between fluid intake, FB and hospital mortality. Multivariate logistic regression models were built as follows: first, variables with a *p* value <0.20 identified by the univariate analysis or that were considered clinically important were included for further multivariable analysis; second, we used a stepwise backward elimination method to remove variables with *p* value >0.2; third, we kept removing and adding variables according to their impact on the coefficient of the other variables until all variables that remained in the model were clinically and statistically important, and the fit of these models were tested using the partial likelihood ratio test [14]. Potential multi-collinearity was tested using the variance inflation factor (VIF), with VIF > =5 indicating the presence of multi-collinearity. Goodness of fit was tested for all logistic regression models. All statistical analysis was performed using the software STATA 11.2 (College Station, TX, USA). All tests were two-sided, and an alpha level of 0.05 was set for statistical significance.

## Results

The MIMIC-II database contains the records of 62,623 admissions of which 42,464 were excluded (16,103 admissions were duplications, 8068 were patients younger than 18 years old and 18,293 spent less than 48 hours in the ICU). Of the remaining 20,159 admissions, 3847 patients were excluded for having undergone cardiac surgery or renal replacement therapy, 11,732 patients were excluded for having positive FB and 2370 patients were excluded for lack of a disease severity score. After removing all the outliers, 2068 patients were included, including 604 non-survivors and 1464 survivors (Table 1), giving a mortality rate of 29.2%. The mean age was 62.3 ± 0.4 years, and 1177 patients were male (56.9%). Both fluid intake and UO were significantly higher in survivors than in non-survivors (53.7 ± 0.7 vs. 48.3 ± 1.0; 67.4 ± 0.8 vs. 60.1 ± 1.3, *p* < 0.001 for both). However, there was no significant difference in FB (*p* = 0.231). Diuretics were used less often in survivors than in non-survivors (31.4% vs. 47.8%, *p* < 0.001).

**Table 1:** Comparisons of baseline characteristics between survivors and non-survivors

Variables	Total (*n* = 2068)	Survivors (*n* = 1464)	Non-survivors (*n* = 604)	*P* value
Age (years)	62.3 ± 0.4	59.3 ± 0.5	69.3 ± 0.5	<0.001
Male (*n* (%))	1177 (56.9%)	817 (55.8%)	360 (59.6%)	0.113
Weight (kg)	82.4 ± 0.5	83.4 ± 0.6	79.8 ± 0.9	<0.001
Reasons for admission				
Cardiac disorder (*n* (%))	627 (30.3%)	405 (27.6%)	222 (36.8%)	<0.001
Cerebral disorder (*n* (%))	367 (17.7%)	282 (19.3%)	85 (14.1%)	0.005
Gastrointestinal disorder (*n* (%))	177 (8.5%)	130 (8.9%)	47 (7.8%)	0.417
Respiratory disorder (*n* (%))	345 (16.7%)	226 (15.4%)	119 (19.7%)	0.018
Sepsis (*n* (%))	1010 (48.8%)	661 (45.1%)	349 (57.8%)	<0.001
Type of ICU				
Medical (*n* (%))	706 (34.1%)	474 (32.3%)	232 (38.4%)	0.009
Cardiac (*n* (%))	547 (26.4%)	346 (23.6%)	201 (33.2%)	<0.001
Surgical (*n* (%))	356 (17.2%)	266 (18.2%)	90 (14.9%)	0.073
AKI incidence (*n* (%))	464 (22.4%)	249 (17.0%)	215 (35.6%)	<0.001
Maximum SOFA score (median (IQR))	6 (3–8)	5 (3–8)	7 (4–10)	<0.001
Fluid balance (ml/kg/48 hours)	–21.4 ± 0.4	–-21.5 ± 0.5	–20.9 ± 0.7	0.231
Fluid intake (ml/kg/48 hours)	52.2 ± 0.6	53.7 ± 0.7	48.3 ± 1.0	<0.001
Urine output (ml/kg/48 hours)	65.3 ± 0.7	67.4 ± 0.8	60.1 ± 1.3	<0.001
Diuretics (*n* (%))	778 (37.6%)	489 (33.4%)	289 (47.8%)	<0.001
Blood products (*n* (%))	490 (23.7%)	300 (20.5%)	190 (31.2%)	<0.001

*AKI* acute kidney injury, *SOFA* sequential organ failure assessment. Diuretic and blood product use was defined as any diuretics or blood products used within 48 hour of ICU admission

**Fig. 1:**
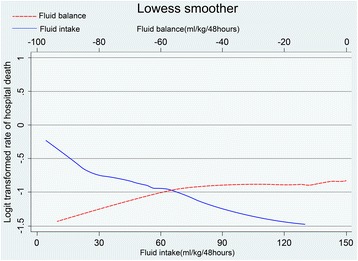
Association between fluid intake/fluid balance and hospital mortality

**Fig. 2:**
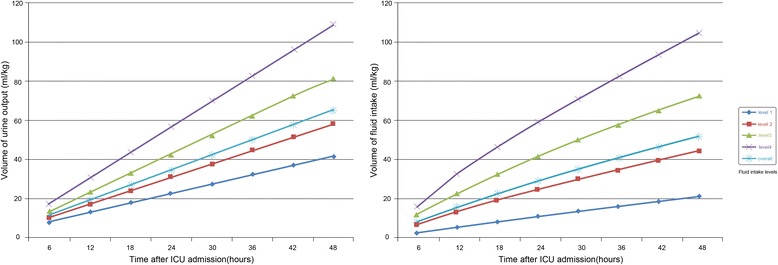
Volume distribution of fluid intake and urine output over the first 48 hours after ICU admission

An approximate negative linear association was observed between fluid intake and hospital mortality using the lowess smoothing technique. No obvious association between FB and hospital mortality was detected except for extreme negative FB (< –50 ml/kg/48 hours) (Fig. 1). Figure 2 shows that the volume of fluid intake and UO was evenly distributed over the 48 hours without obvious fluctuation.

Crude outcomes by four fluid intake levels are displayed in Table 2. Hospital mortality was decreased stepwise from level 1 (34.9%) to level 4 (20.8%) (*p* < 0.001). The SOFA score showed no significant difference in disease severity within any of the four levels, while the SAPS II score was higher in level 4 (*p* < 0.001). Diuretics were used less in level 3 and 4 than in level 1 and 2, while blood products were used more in the higher levels. Of note, there was only a slight difference in FB despite the big gap in fluid intake volume in the four fluid intake levels.

**Table 2:** Characteristics and outcomes by fluid intake categories

	Fluid intake categories (ml/kg/48 hours)				
Outcomes	<29 (*n* = 438)	30 ~ 59 (*n* = 941)	60 ~ 89 (*n* = 482)	>90 (*n* = 207)	*P* value
Hospital mortality (*n* (%))	153 (34.9%)	289 (30.7%)	116 (24.1%)	43 (20.8%)	<0.001
ICU LOS (median (IQR))	3.6 (5.3–13.6)	3.7 (2.7–6.0)	3.5 (2.7–5.9)	4.0 (2.8–9.7)	0.023
AKI (*n* (%))	141 (32.2%)	226 (24.0%)	67 (13.9%)	30 (14.5%)	<0.001
Maximum SOFA score (median (IQR))	6 (4–9)	6 (3–8)	6 (3–8)	7 (4–9)	0.454
Maximum SAPS (median (IQR))	14 (11–17)	14 (11–17)	14 (12–18)	16 (10–19)	<0.001
Diuretics (*n* (%))	202 (46.1%)	416 (44.2%)	124 (25.7%)	36 (17.3%)	<0.001
Blood products (n (%))	50 (11.4%)	231 (24.5%)	144 (29.8%)	65 (31.4%)	<0.001
Intravenous fluid intake (ml/kg/48 hours)	13.3 ± 0.4	34.0 ± 0.4	63.5 ± 0.7	93.7 ± 1.2	<0.001
Oral fluid intake (ml/kg/48 hours)	8.2 ± 0.3	10.3 ± 0.3	9.1 ± 0.5	11.0 ± 1.0	<0.001
Fluid balance (ml/kg/48 hours)	–25.4 ± 19.0	–21.4 ± 18.1	–18.5 ± 15.9	–19.4 ± 18.1	<0.001

*LOS* length of stay, *AKI* acute kidney injury, *SOFA* sequential organ failure assessment, *IQR* interquartile range, *SAPS* simplified acute physiology score. Diuretic and blood product use were defined as any diuretics or blood product used within 48 hour of ICU admission. *P* value represents the overall statistical difference within the four categories

Univariate analysis (Table 3) showed that both increased fluid intake and UO were associated with lower hospital mortality, while FB was insignificant. Use of both blood products and diuretics was associated with higher mortality (*p* < 0.001 for both). After adjusting for covariates, the outcomes were similar to those in univariate analysis. Using level 1 as the reference group, increased fluid intake was associated with decreased hospital mortality (Table 4), with the odds ratio (OR) decreased stepwise from level 2 (OR 0.73, 95% CI 0.56–0.96) to level 4 (OR 0.47, 95% CI 0.30–0.74). Compared to a slight negative FB (level 1), there was a decreased tendency towards mortality with FB level 2 (OR 0.88, 95% CI 0.69–1.11) and level 3 (OR 0.79, 95% CI 0. 65–1.11); however, only extreme negative FB (level 4) was significant (OR 0.56, 95% CI 0. 33–0.95). We also built two logistic models for FB and fluid intake using the linear spline function with a knot detected (Fig. 1), and the results showed a similar pattern (see Additional file 1). UO was also negatively associated with hospital mortality in these patients (see Additional file 1).

**Table 3:** Univariate logistic regression analysis for hospital mortality

Variables	Crude odds ratio	95% Confidence interval	*P* value
Fluid balance (ml/kg/48 hours)			
Level 1 (–19 ~ 0)	1.00	-	-
Level 2 (–39 ~ –20)	1.00	0.81–1.25	0.942
Level 3 (–59 ~ −40)	0.98	0.71–1.34	0.901
Level 4 (< −60)	0.77	0.48–1.26	0.309
Fluid intake (ml/kg/48 hours)			
Level 1 (<29)	1.00	-	-
Level 2 (30 ~ 59)	0.83	0.65–1.04	0.118
Level 3 (60 ~ 89)	0.59	0.44 – 0.78	<0.001
Level 4 (>90)	0.53	0.36 – 0.78	0.001
Intravenous fluid intake (ml/kg/48 hours)			
Level 1 (<29)	1.00	-	-
Level 2 (30 ~ 59)	0.81	0.65–1.00	0.005
Level 3 (60 ~ 89)	0.64	0.48–0.84	0.001
Level 4 (>90)	0.57	0.37–0.87	0.009
Urine output (ml/kg/48 hours)			
Level 1 (<29)	1.00	-	-
Level 2 (30 ~ 59)	0.59	0.43–0.80	0.001
Level 3 (60 ~ 89)	0.48	0.35–0.67	<0.001
Level 4 (>90)	0.36	0.25–0.52	<0.001
Blood products	1.80	1.51–2.22	<0.001
Diuretics	1.78	1.44–2.20	<0.001

**Table 4:** Adjusted odds ratio using fluid intake and fluid balance as design variables in multivariable logistic regression

Model 1				Model 2			
Variables	Odds ratio	95% CI	*P* value	Variables	Odds ratio	95% CI	*P* value
Fluid intake level 1 (≤29)	Reference			Fluid balance level 1 (–19 ~ 0)	Reference		
Level 2 (30 ~ 59)	0.73	0.56–0.96	0.024	Level 2 (–39 ~ –20)	0.88	0.69–1.11	0.286
Level 3 (60 ~ 89)	0.61	0.43–0.85	0.004	Level 3 (–59 ~ –40)	0.79	0. 65–1.11	0.181
Level 4 (>90)	0.47	0.30–0.74	0.001	Level 4 (≤ –60)	0.56	0. 33–0.95	0.034
Diuretics	1.36	1.09–1.68	0.005	Diuretics use	1.50	1.21–1.86	<0.001
Blood products	1.51	1.19–1.91	0.001	Blood product	1.36	1.08–1.72	0.009
Weight	0.98	0.98–0.99	<0.001	Weight	0.99	0.98–0.99	<0.001
Maximum WBC	1.03	1.02–1.05	<0.001	Maximum WBC	1.03	1.01–1.04	<.0001
Maximum SOFA score	1.11	1.08–1.14	<0.001	Maximum SOFA	1.11	1.07–1.13	<0.001
Maximum serum creatinine	1.15	1.08–1.22	<0.001	Maximum serum creatinine	1.18	1.11–1.26	<0.001
MICU	1.74	1.35–2.24	<0.001	MICU	1.89	1.48–2.42	<0.001
CCU	2.11	1.61–2.77	<0.001	CCU	2.29	1.75–2.99	<0.001

*WBC* white blood cells, *SOFA* sequential organ failure assessment, *MICU* medical ICU, *CCU* coronary care unit. Diuretic and blood product use was defined as any diuretics or blood products used within 48 hour of ICU admission while maximum serum creatinine and SOFA values are for the whole ICU stay. The mean variance inflation factor was 2.44 and 2.15 and the *p* value for goodness of fit was 0.258 and 0.372 in model 1and model 2, respectively

## Discussion

To the best of our knowledge, this is the first study investigating fluid management in critically ill patients with negative FB. Our results suggest that both increased fluid intake and UO are associated with decreased hospital mortality in these patients. However, compared to a slight negative FB, achieving a more negative FB was not associated with reduced mortality except for an extreme negative FB (≤ –60 ml/kg/48 hours).

The impact of negative FB on clinical outcomes has been described in several specific populations. Alsous et al. [15] demonstrated that mortality is significantly increased in patients with septic shock who do not achieve negative FB within 3 days of treatment, which is consistent with Boyd’s findings [3]. In a multicenter cohort study, Payen et al. [4] reported that positive FB is strongly associated with increased 60-day mortality in patients with acute renal failure. Similar results are also confirmed in other disease categories, such as ASH [16] and cancer [17], and in critically ill surgical patients [7, 18].

However, the current evidence focused on comparison between positive and negative FB, and whether achieving a more negative FB could further improve clinical outcomes in patients with negative FB remains unclear. Sakr et al. [6] reported that a more negative FB (up to 7 days) is associated with better outcomes in patients with ASH (–0.7 L vs. –5.6 L, *p* < 0.001). However, this comparison between a slight and a more negative FB may have been influenced by the inclusion of patients with positive FB.

In the current study there was no significant association between the degree of negative FB and hospital mortality, except with an extreme negative FB (≤ –60 ml/kg/48 hours). The underlying mechanism remains unclear. However, the ability to achieve a negative FB may be an important signal of the stabilization of the hemodynamic conditions, and of the recovery of cardiac and renal functions. Thus, we concluded that within a certain range, the degree of negative FB only depends on the previous extent of fluid accumulation instead of organ function. If this is the case, a strategy aiming at achieving a more negative FB would not be suitable for these patients. However, the ability to achieve an extreme negative FB may suggest better cardiac and renal function, and it was associated with a lower death rate.

On the other hand, restricted fluid management [5, 19, 20] has been widely adopted in clinical practice once hemodynamic stability is achieved, aiming to avoid fluid overload, which has been strongly associated with adverse outcomes [3, 9, 21]. However, whether restricted fluid strategy still applies to patients with negative FB has not been studied.

In the present study, we found that both fluid intake and UO were negatively associated with hospital mortality, which suggests that restricted fluid management was no longer suitable for these patients. However, the interrelationship between them was changeable and complicated. Two important relationships should be addressed. The first one is the relationship between fluid intake and UO. For example, in patients suffering from severe fluid overload or organ dysfunction, increased UO may be a recovery sign, and more fluid may be given to these patients. Under this condition, the association between fluid intake and mortality is only due to selection bias instead of there being a causal relationship. However, we believe this possibility was relatively low in our study due to two reasons: first, this is a highly select group of patients, and the ability to achieve negative FB may represent more stable hemodynamics and compensated organ function (mainly cardiac and kidney function), at least compared to patients with positive FB; second, we found that both volume of fluid intake and UO were evenly distributed, with a constant rate over the first 48 hours after ICU admission (Fig. 2) and there was strong correlation between them (Pearson correlation, *r* = 0.69, *p* < 0.001). Thus, we believe that much of the fluid administration was protocol-driven and not given therapeutically for confounding factors such as hypotension and blood loss. Thus it is more likely that fluid intake was the leading cause of increased UO in these patients and the association between UO and mortality is a secondary result of association between fluid intake and mortality in this population instead of there being a causal relationship. This conclusion led to our next question: what caused this association between fluid intake and mortality? We noticed that the difference in FB was small within four fluid intake levels (Table 2). One explanation is that the ability to achieve a similar negative FB volume under a higher dose of fluid intake may suggest better organ function. However, the SOFA score did not differ within the four fluid intake levels and the SAPS II score was even higher in level 4. Based on all this information, we hypothesized that there may be a causal link between increased fluid intake and decreased mortality in this specific population (Table 2), which could also partly explain the inconsistent results of studies focusing on the comparison between conservative and liberal fluid management [3, 4, 9, 22–25]. However, the mechanism could not be inferred from our study. Whether it is correlated with improved homeostasis such as balance of the internal environment (electrolyte), the excretion of inflammatory factors still needs to be further investigated.

Administration of diuretics in the critically ill population continues to be controversial, particularly in regard to which patients will benefit from them, the optimal time point to initiate diuretics and the most appropriate end point for therapy. The potential risks of diuretic administration in the ICU population are substantial, including hemodynamic compromise, electrolyte abnormalities and renal dysfunction. In the current study, diuretic use was associated with increased mortality in these patients. However, it is difficult to conclude a causal relationship between them as this could be a surrogate for edema or other factors.

The main advantage of the present study is the large sample size. The size of the cohort meant it was possible to perform this analysis despite the small proportion of patients with negative FB. However, the study also had several limitations. First, fluid balance prior to ICU admission was disregarded due to limited information in the database, which to a certain degree could lead to information bias. Second, patients who underwent cardiac surgery or kidney replacement therapy were excluded. Thus, the findings of the present study are not generalizable to these patients. Third, the choice of 48 hours as the critical time point to assess whether or not a patient achieved negative FB was arbitrary. However, the cutoff period varies across studies and no consensus has been achieved. Fourth, only loop diuretics were included in our study, and the association between mortality and other diuretics such as acetazolamide still needs investigation. Finally, due to the nature of retrospective research, only the association between fluid intake and mortality could be inferred. Despite this, several valid hypotheses have been made in our study that may provide effective evidence for further research to establish a definitive causal link.

## Conclusion

In conclusion, by the analysis of a large clinical database, our study shows that both increased fluid intake and UO were associated with decreased mortality in patients with negative FB. However, compared to a slight FB, only an extreme degree of FB was associated with reduced mortality. Further studies are needed to investigate the causal relationship and mechanism underlying this association.

## Additional file

Additional file 1: Table S1. Multivariable logistic regressions of fluid intake and fluid balance using linear spline function. **Table S2.** Adjusted odds ratio using urine output as design variables in multivariable logistic regression. **Table S3.** Volume distribution of fluid intake and urine output during the first 48 hours after ICU admission. (DOCX 25 kb)

## Abbreviations

AKI: Acute kidney injury
ANOVA: Analysis of variance
ASH: Aneurysmal subarachnoid hemorrhage
FB: Fluid balance
LOS: Length of stay
SAPS: Simplified acute physiology score
SOFA: Sequential organ failure assessment
UO: Urine output
VIF: Variance inflation factor
