# Supplementary material for: Association between fluid intake and mortality in critically ill patients with negative fluid balance: a retrospective cohort study
Source: Crit Care. 2017 May 12;21:104. doi: 10.1186/s13054-017-1692-3 (PMC5427534; doi:10.1186/s13054-017-1692-3)
Supplement: Additional file 1: Table S1. — Multivariable logistic regressions of fluid intake and fluid balance using linear spline function. Table S2. Adjusted odds ratio using urine output as design variables in multivariable logistic regression. Table S3. Volume distribution of fluid intake and urine output during the first 48 hours after ICU admission. (DOCX 25 kb) [file 13054_2017_1692_MOESM1_ESM.docx]

| Model s1 | | | | |
| --- | --- | --- | --- | --- |
| Variables | **Crude Odds ratio** | **p** | **Adjusted Odds ratio** | **p** |
| Fluid balance (< -50 ml/kg/48hours) | 1.014 | 0.225 | 1.016 | 0.193 |
| Fluid balance (-49 ~ 0 ml/kg/48hours) | 0.999 | 0.889 | 1.006 | 0.087 |
| Model s2 | | | | |
| Variables | Crude Odds ratio | p | Adjusted Odds ratio | p |
| Fluid intake (0 ~ 29 ml/kg/48hours) | 0.983 | 0.136 | 0.973 | 0.036 |
| Fluid intake (> 30 ml/kg/48hours) | 0.993 | 0.002 | 0.992 | 0.011 |

Table S1 multivariable logistic regressions of fluid intake and fluid balance using linear spline function.

Note: The mean VIFs were 4.91 and 4.71 and p values of goodness of fit were 0.364 and 0.262 in model 1 and model 2, respectively.

Both two models were adjusted for, weight, maximum SOFA score, maximum serum creatinine, maximum WBC, blood products and diuretics use, type of admitted ICU.

Table S2 Adjusted odds ratio using urine output as design variables in multivariable logistic regression.

| Model 3 | | | |
| --- | --- | --- | --- |
| Variables | **Odds ratio** | **95% CI** | **p** |
| Urine output level 1 (< 29) | Ref. | | |
| Level 2 (30 ~ 59) | 0.74 | 0.52 – 1.06 | 0.100 |
| Level 3 (60 ~ 89) | 0.53 | 0.36 – 0.78 | 0.001 |
| Level 4 (> 90) | 0.39 | 0.26 – 0.61 | < 0.001 |
| Diuretics | 1.39 | 1.12 – 1.72 | 0.003 |
| Blood products | 1.41 | 1.11 – 1.78 | 0.004 |
| Age | 1.003 | 1.00 – 1.01 | < 0.001 |
| Weight | 0.99 | 0.98 – 0.99 | < 0.001 |
| Maximum WBC | 1.03 | 1.02 – 1.04 | < 0.001 |
| Maximum SOFA | 1.11 | 1.08 – 1.14 | < 0.001 |
| Maximum serum creatinine | 1.12 | 1.08 – 1.22 | 0.001 |
| MICU | 1.76 | 1.38 – 2.27 | < 0.001 |
| CCU | 2.11 | 1.62 – 2.77 | < 0.001 |

Note: The mean variance inflation factor (VIF) was 2.91 and p value of goodness of fit was 0.418.

Table S3 Volume distribution of fluid intake and urine output during the first 48 hours after ICU admission

|  | Fluid intake categories (ml/kg/48hours) | | | | |
| --- | --- | --- | --- | --- | --- |
| Outcomes | < 29 (n = 466) | 30 ~ 59 (n = 1026) | 60 ~ 89(n = 557) | > 90(n = 349) | Overall |
| Fluid intake (ml/kg/6hours) | 3.1 ± 3.3 | 7.2 ± 6.3 | 12.3 ± 9.9 | 16.5 ± 12.8 | 8.4 ± 8.7 |
| Fluid intake (ml/kg/12hours) | 6 ± 4.2 | 13.9 ± 7.7 | 23.3 ± 11.6 | 32.9 ± 15.9 | 16.3 ± 12.3 |
| Fluid intake (ml/kg/18hours) | 8.8 ± 4.7 | 19.8 ± 8.5 | 32.9 ± 11.8 | 46.5 ± 16.7 | 23.2 ± 14.9 |
| Fluid intake (ml/kg/24hours) | 11.6 ± 4.9 | 25.1 ± 8.6 | 42 ± 11.7 | 59.2 ± 15.7 | 29.5 ± 17.2 |
| Fluid intake (ml/kg/30hours) | 14.0 ± 5.1 | 30.1 ± 8.8 | 50.1 ± 9.8 | 71.3 ± 14.9 | 35.3 ± 19.5 |
| Fluid intake (ml/kg/36hours) | 16.4 ± 5.3 | 34.7 ± 8.6 | 57.7 ± 10.7 | 82.813.6 | 41 ± 21.6 |
| Fluid intake (ml/kg/42hours) | 18.8 ± 5.4 | 39.5 ± 8.5 | 65.3 ± 8.8 | 93.4 ± 11.5 | 46.5 ± 23.6 |
| Fluid intake (ml/kg/48hours) | 21.5 ± 5.8 | 44.4 ± 8.5 | 72.6 ± 8.3 | 104.8 ± 10.9 | 52.1 ± 25.8 |
| Urine output (ml/kg/6hours) | 7.9 ± 7.2 | 10.2 ± 8.6 | 13.7 ± 10.2 | 17.4 ± 12.9 | 11.2 ± 9.6 |
| Urine output (ml/kg/12hours) | 13.2 ± 10 | 17.2 ± 11.6 | 23.2 ± 12.7 | 30.9 ± 16.6 | 19.2 ± 13.2 |
| Urine output (ml/kg/18hours) | 18.2 ± 12.2 | 24.2 ± 14.1 | 32.9 ± 15.4 | 43.9 ± 18.7 | 26.9 ± 16.4 |
| Urine output (ml/kg/24hours) | 23.1 ± 13.9 | 31.2 ± 15.9 | 42.5 ± 17.4 | 57.0 ± 2.9 | 34.7 ± 19.2 |
| Urine output (ml/kg/30hours) | 28.1 ± 15.9 | 38.1 ± 17.5 | 52.4 ± 18.6 | 70.3 ± 22.8 | 42.5 ± 21.8 |
| Urine output (ml/kg/36hours) | 32.9 ± 17.9 | 44.9 ± 19.2 | 62.3 ± 20.1 | 82.9 ± 24.2 | 50.2 ± 24.6 |
| Urine output (ml/kg/42hours) | 37.3 ± 19.5 | 51.7 ± 21.1 | 72.9 ± 21.9 | 96.8 ± 25.6 | 57.9 ± 27.7 |
| Urine output (ml/kg/48hours) | 41.9 ± 21.4 | 58.2 ± 22.9 | 81.3 ± 23.4 | 109.2 ± 27.3 | 65.3 ± 30.5 |
